# Correction: MultipleTesting.com: A tool for life science researchers for multiple hypothesis testing correction

**DOI:** 10.1371/journal.pone.0274662

**Published:** 2022-09-09

**Authors:** Otília Menyhart, Boglárka Weltz, Balázs Győrffy

After the publication of this article [1] it was noticed that in the Sequential methods for multiple-testing correction subsection of the Introduction, the description of the Hochberg-correction contains an error.

The correct paragraph is: The Hochberg-correction, also called the step-up method, is based on a reverse scenario when the largest p-value is examined first. Once a significant p-value is identified, all the remaining smaller p-values would be declared significant (13). For example, if n = 500, the largest p-values are 0.3, 0.05, 0.01, and α = 0.05, the following adjustments are concluded:

Rank#1: 0.3 * 1 = 0.3, 0.3 > 0.05, the test is not significant
Rank#2: 0.05 * 2 = 0.1, 0.1 > 0.05, the test is not significant
Rank#3: 0.01 * 3 = 0.03, 0.03 < 0.05, the test is significant, reject the hull hypothesis, and all of the remaining p-values will be significant after correction.

The authors have stated that the online tool developed for multiple hypothesis testing corrections at www.multipletesting.com employs the correct Hochberg step-up algorithm.
