# Supplementary material for: MultipleTesting.com: A tool for life science researchers for multiple hypothesis testing correction
Source: PLoS One. 2021 Jun 9;16(6):e0245824. doi: 10.1371/journal.pone.0245824 (PMC8189492; doi:10.1371/journal.pone.0245824)
Supplement: Attachment — Submitted filename: Rebuttal letter 20210426_b.docx [file pone.0245824.s004.docx]

**Dear Editor-in-Chief,**

Thank you for the opportunity to submit a revised version of our manuscript.

Please find below our point-by-point responses to the issues raised by the reviewers (the implemented changes are also highlighted in the manuscript):

*Comments from the Editor:*

In the revised version of the paper please address the reviewers' comments listed at the end of this email. Additionally, please provide a comprehensive literature review in which you point out similar works in terms of tools development and works that use the type of analysis for which the current tool has been developed. Please carefully check the components presented in Figure 1. Also, please provide a real life science example in which the use of this tool will ease the work of the researches. Please provide the limitations of the study and point out the possible directions of extending the work. Finally, please compare the facilities offered by your tool with other similar tools available online.

**Thank you for your precious input. We have addressed every comment raised by the reviewers and have extended our literature review by recent articles and tools developed for controlling multiplicity in high throughput data, a particularly rapidly growing field of science. We have substantially expanded the number of cited articles. We have also addressed the strategy illustrated in Figure 1. We are discussing examples for real-life applications, where controlling for multiplicity is a common issue.**

**The topic of multiple hypothesis testing is far-reaching and rapidly proliferating, and our manuscript is limited to the introduction of the most extensively utilized methods. Another limitation is the lack of detailed description of technical parts of the presented methods, although the provided examples clarify the concepts. We articulate our goals more clearly in the revised version of the manuscript.**

**We have conducted an extensive literature search but could not locate a similar user-friendly, web-based tool for conducting multiple hypothesis testing without the need for programming knowledge. We now include a section about tools implemented in R, and emphasize the facilities enabled by our platform. Our goal for the future is to extend the repertoire of available methods further, which we also highlight in the manuscript.**

*Reviewer comments:*

**Reviewer #1:** The authors provide a review of the most frequently used approaches for correction methods in multiple hypothesis testing.
The simultaneous problem, introduced as early as the mid-twentieth century, is interesting and has been recently revived due to advanced in technology. However, I think the authors miss to give a contribution to the topic. There are several reviews on the subject which I believe are complete. [1], [2] and [3] are examples of extensive surveys over correction methods.

1. Stefanie R Austin, Isaac Dialsingh, and Naomi Altman. Multiple hypothesis testing: A review. J. Indian Soc. Of Agricultural Stat, 68:303–314, 2014.
2. Farcomeni A. A review of modern multiple hypothesis testing, with particular attention to the false discovery proportion. Statistical Methods in Medical Research, 17:347 (88), 2007.
3. Shaffer, J. P. (1995). Multiple hypothesis testing. Annual Review of Psychology, 46(1), 561–584.

**Thank you very much for the valuable suggestions. Following the reviewer's advice, we incorporated a section about additional, more recent methods, focusing mainly on novel FDR-controlling strategies. We have also included some of the suggested publications, complemented by additional comprehensive reviews, to enhance our contribution to the topic; please see pages 10-12.**

**Also, after reviewing the scientific literature for procedures and tools available for multiple testing, we could only identify tools requiring programming skills and the use of a command line. Although programming is increasingly important, this knowledge is not yet a default state among scientists. We feel that our online calculator accessible at** [**www.multipletesting.com**](http://www.multipletesting.com) **is our main contribution and fills a gap by not only providing a user-friendly option for researchers but also directly enabling the comparison of the results of the most widely used methods.**

Reviewer #2: The study "MultipleTesting.com: a tool for life science researchers for multiple hypothesis testing correction" is interesting. To facilitate multiple-testing corrections, the authors developed a fully automated solution not requiring programming skills or the use of a command line. The current research provides a much needed practical synthesis of basic statistical concepts regarding multiple hypothesis testing in a comprehensible language with well-illustrated examples. The web tool will fill the gap for life science researchers by providing a user-friendly substitute for command-line alternatives. The paper is well set, and the problem highlighted executed properly. However, attention should be given to the following highlighted points before resubmitting.

**Thank you very much for the positive remarks regarding our manuscript.**

1. Page 10 of 22, "The procedure results in a statistical confidence measure, called a p-value, compared to the level of significance, α. When p is smaller than the confidence threshold α, the null hypothesis is rejected with a certain confidence, but the rejection does not "prove" the alternative hypothesis." The P-value is called the observed significance level than why we are comparing with α. Why not take a straight level of significance from the P value. For example, if P = 0.11, means the significance level is 11%.

**Thank you for your suggestion. P-values indicate how extreme the data are, while alpha values determine how extreme the data must be before the null hypothesis is rejected. We have extended our description and clarified the concept further; please see page 4. We feel that our description helps the readers by showing the difference between these two concepts.**

2. In the last, more recent references should be added to broaden the view of readers and enhance the new contribution of this paper for comparison.

**Thank you for the suggestion. According to the reviewer's request, we have incorporated additional work to enhance our contribution to the topic and to broaden the view of our readers, focusing mainly at recent FDR-controlling methods; please see pages 10-12.**

3. The authors needed to add some more explanation in the conclusion section.

**Thank you for your observation. We have incorporated additional examples, mainly aimed at life scientists, and expanded our Conclusions section; please see pages 12-13.**

Reviewer #3: The authors developed an online tool for life science researchers for multiple hypothesis testing corrections. The online tool compiles the five most frequently used adjustment tools which can enable researchers to calculate False Discovery Rates (FDR) and q-values for multiple-testing corrections.

I suggest the author check the citation on page 9, paragraph 3, line 2 for proper citation.

**Thank you for your remark, we have corrected the citation; please see page 9.**

I suggest moving the supplemental Table 1 to the main work due to its relevance.

**Thank you for your suggestion; we now moved Table 1 to the main body of the manuscript.**

Provide a friendly user-guide in the online tool to make it easier and more useful to targeted research communities.

**Thank you for your recommendation. We have incorporated a short description into the online platform to enhance its practicality.**

The paper presented original research, reported in standard English with relevance literature discussed. With the level of contribution presented by the authors, I, therefore, recommend the manuscript for consideration by PLOS ONE after minor revision.

**We appreciate the positive evaluation and have edited the manuscript to increase its standards further.**

**We hope that the issues raised about the manuscript have been sufficiently addressed in this improved version. On this occasion, we would also like to thank the Editor and the three anonymous reviewers for their expert and helpful comments.**

With best regards:

Balázs Győrffy MD PhD
